# Missed opportunities in tuberculosis investigation and associated factors at public health facilities in Uganda

**DOI:** 10.1186/s12913-021-06368-6

**Published:** 2021-04-17

**Authors:** Keith Twirire Kakame, Noel Namuhani, Andrew Kazibwe, Felix Bongomin, Joseph Baruch Baluku, Sebastian Olikira Baine

**Affiliations:** 1 Department of Medicine, College of Health Sciences, Makerere University, P.O Box 7072, Kampala, Uganda; 2 School of Public Health, College of Health Sciences, Makerere University, P.O Box 7072, Kampala, Uganda; 3 The AIDS Support Organization (U) Ltd, P.O Box 10443, Kampala, Uganda; 4 Department of Medical Microbiology & Immunology, Faculty of Medicine, Gulu University, P.O Box 166, Gulu, Uganda; 5 Division of Pulmonology, Kiruddu National Referral Hospital, P.O Box 7272, Kampala, Uganda; 6 Directorate of Programs, Mildmay Uganda, P.O Box 24985, Kampala, Uganda

**Keywords:** Missed opportunities, Tuberculosis case finding, Patient factors, Uganda

## Abstract

**Background:**

The incidence of tuberculosis (TB) is high in Uganda; yet, TB case detection is low. The population-based survey on the prevalence of TB in Uganda revealed that only 16% of presumptive TB patients seeking care at health facilities were offered sputum microscopy or chest-X ray (CXR). This study aimed to determine the magnitude of, and patient factors associated with missed opportunities in TB investigation at public health facilities of Wakiso District in Uganda.

**Methods:**

A facility-based cross-sectional survey was conducted at 10 high volume public health facilities offering comprehensive TB services in Wakiso, Uganda, among adults (≥18 years) with at least one symptom suggestive of TB predefined according to the World Health Organisation criteria. Using exit interviews, data on demographics, TB symptoms, and clinical data relevant to TB diagnosis were collected. A missed opportunity in TB investigation was defined as a patient with symptoms suggestive of TB who did not have sputum and/or CXR evaluation to rule out TB. Poisson regression analysis was performed to determine factors associated with missed opportunities in TB investigation.

**Results:**

Two hundred forty-seven (247) patients with presumptive TB exiting at antiretroviral therapy (ART) clinics (*n* = 132) or general outpatient clinics (*n* = 115) at public health facilities were recruited into this study. Majority of participants were female (161/247, 65.2%) with a mean + SD age of 35.1 + 11.5 years. Overall, 138 (55.9%) patients with symptoms suggestive of TB disease did not have sputum and/or CXR examinations. Patients who did not inform health workers about their TB related symptoms were more likely to miss a TB investigation (adjusted prevalence ratio (aPR): 1.68, 95%CI; 1.36–2.08, *P* < 0.001). However, patients who reported duration of cough of 2 weeks or more were less likely to be missed for TB screening (aPR; 0.69, 95%CI; 0.56–0.86, *p* < 0.001).

**Conclusion:**

There are substantial missed opportunities for TB diagnosis in Wakiso District. While it is important that patients should be empowered to report symptoms, health workers need to proactively implement the WHO TB symptom screen tool and complete the subsequent steps in the TB diagnostic cascade.

## Introduction

Tuberculosis (TB) is a global public health problem and a leading cause of death from an infectious agent [1]. In 2018, it was estimated that there were 10 million new TB cases worldwide and 1.5 million TB deaths [1]. About 24% of these TB cases were in sub-Saharan Africa which accounts for about 86% of the global TB/HIV burden [1].

The United Nation’s Sustainable Development Goals (SDGs) and the End TB strategy aim to end TB by 2030 [2]. Early diagnosis of TB is a critical component of the End TB interventions [2]. Despite roll out of new diagnostic tools such as GeneXpert to improve diagnosis, TB cases are still missed globally. In 2018, about 3 million people with TB were either missed or not reported and only one in three people with drug resistant TB accessed care [1]. Missed TB cases result in delayed treatment and premature death, complications, community and nosocomial transmission, and catastrophic costs for families [3, 4].

Uganda National TB and Leprosy Control Program recommends prompt screening for TB among all persons seeking health care at healthcare facilities [5, 6]. However, the population based survey on the prevalence of TB in Uganda found that only 16% of patients with symptoms suggestive of TB who visited health facilities were investigated for TB by sputum microscopy or Chest X-ray (CXR) [7]. Similar observations were reported from South Africa and India [8–10]^.^

Previous studies on factors associated with missed TB cases in health facilities revealed that: health system factors such as lack of training, low staff motivation, and high workload; and contextual factors including time and cost borne by patients to seek and complete TB evaluation, poor health literacy, and stigma against patients contribute to missed opportunities in TB investigation at public health facilities [11].

In South Africa, a study done at clinics participating in a cluster randomised trial found that patient factors associated with TB investigation include increasing number of symptoms such as longer duration of cough, unintentional weight loss, and night sweats and reporting symptoms to healthcare worker [8].

There is a dearth of knowledge on patient factors associated with missed TB investigation among adults with TB related symptoms in public health facilities in resource limited settings. The objective of this study was to determine the magnitude of, and patient factors associated with missed opportunities in TB investigation at public health facilities in a peri-urban district in Central Uganda.

## Methods

### Study design and setting

This was a cross-sectional study conducted between April and June 2018 among adults who presented with symptoms suggestive of TB predefined according to the WHO criteria. Patients exiting 10 high volume public health facilities in Wakiso district in Uganda constituted the study population. In 2014, Wakiso District had an estimated population of 2.0 million [12]. About 60% of this population live in the urban areas [12]. Since 2010, interventions have been implemented to improve the case detection rate in the district. The interventions included: the DETECT child TB project; and roll out of national TB/HIV guidelines [13]. However, TB case detection rate remain low at about 57% [13].

Wakiso district has seven health sub districts namely Entebbe, Busiro South, Busiro North, Busiro East, Kyadondo North, Kyadondo South, and Kyadondo East. It hosts 67 public health facilities that offer free comprehensive primary health care services including screening and testing for TB. TB screening and testing services are expected to be offered at all care entry points especially outpatient, HIV/ART clinic, and Maternal & Child Health departments.

### Study population

The study population included adults exiting the public health facilities at two care entry points; HIV/ART clinic and outpatient department. We included all adults aged 18 years or older with at least one symptom suggestive of TB predefined according to WHO criteria (namely - cough lasting for 2 weeks or more, night sweats, unintentional weight loss, and fever). In addition, for people living with HIV, we included patients presenting with cough of any duration. Patients who had sputum sent for TB investigation prior to current visit and TB patients who were already on TB treatment were excluded from the study and so were patients who were deemed incompetent to provide an informed written consent. In addition, patients with symptoms of TB other than those predefined by WHO criteria were also excluded from this study.

### Sample size

We calculated a sample size of 255 clients using Kish (1965) formula for cross sectional studies [14]. According to a study to evaluate TB diagnostic practices at five primary care health facilities in Uganda for 1 year, proportion of patients with symptoms suggestive of TB offered sputum examination was 21% [15]. Hence *p* = 0.21, q = 0.79, d (acceptable degree of error) =0.05, z (standard normal value corresponding to 95% confidence interval) =1.96.

$$ N=\frac{Z^2p\left(1-p\right)}{d^2} $$

Therefore $$ N=\frac{(1.96)^2x\ (0.21)x\ (0.79)}{(0.05)^2} $$ = 255 participants.

### Sampling procedure

Four health sub-districts were randomly selected from the seven health sub districts in Wakiso district. We then purposively selected 10 high volume health centres from the four health sub districts. They included Entebbe Hospital, Kasangati Health Centre IV, Wakiso Health Centre IV, Kajjansi Health centre IV, Buwambo Health Centre IV, Bweyogerere Health Centre III, Kiira Health Centre III, Nabweru Health centre III, Nsangi Health centre III, and Nakawuka Health Centre III. Each high-volume facility received an average of 98 (range 61–160) patients per day. The number of patients to be interviewed at each facility was determined by proportionate to size sampling. This depended on the average number of daily outpatient attendance over the last 3 months.

### Data collection

Patients were screened consecutively for interviews as they exited the different clinicians’ rooms at OPD and HIV clinics. An interviewer-administered structured questionnaire was used to collect data on demographics, TB symptoms, and other clinical data relevant to TB diagnosis. Participants were also asked if they had sputum and/ or a CXR requested by a healthcare worker at that visit. If sputum and/ or CXR had not been requested, they were referred back to the clinic staff for appropriate investigations.

### Quality control

The questionnaire used was pretested in two public health facilities and these were not part of the study sample. Research assistants were trained and supervised during data collection. Filled questionnaires were reviewed daily to check for completeness and consistency.

### Data analysis

Data were entered in Epidata version 3.1 database (EpiData database, Odense, Denmark). Data were cleaned and exported to Stata v14 (StataCorp LP, College Station, TX, USA) for analysis.

Continuous variables were described using means, or medians and the corresponding standard deviations or the interquartile ranges respectively while categorical variables were described using frequencies and percentages. The proportion of missed opportunities in TB investigation was calculated by dividing the number of patients with symptoms suggestive of TB who did not have sputum examination and/or CXR requested to rule out TB disease by the total number of patients with symptoms suggestive of TB disease.

At bivariate analysis, modified Poisson regression was used to identify factors significantly associated with missed TB diagnosis. A *p* < 0.05 was used as level of significance at the 95% confidence interval (95% CI) to test this association. Prevalence ratio was used as the measure of association. At multivariate analysis, factors associated with the primary outcome at bivariate analysis were included in a multivariable model and adjusted prevalence ratios and 95% CI were estimated. A *p* < 0.2 was used as a cut off to determine which variables to carry for multiple modified Poisson regression model to build the final model. Forward regression technique was used to build the multiple modified Poisson regression model while assessing the model variables for significance at *p* < 0.05 and 95% CI. An adjusted R2 was generated for the final model to determine to what extent the factors were associated with the outcome of interest.

### Ethical considerations

Makerere University School of Public Health Research and Ethics Committee (FWA00011353) provided ethical approval and Wakiso District Health Office provided approval and permission to perform the study in the public health facilities.

## Results

### Characteristics of the respondents

**Fig. 1:**
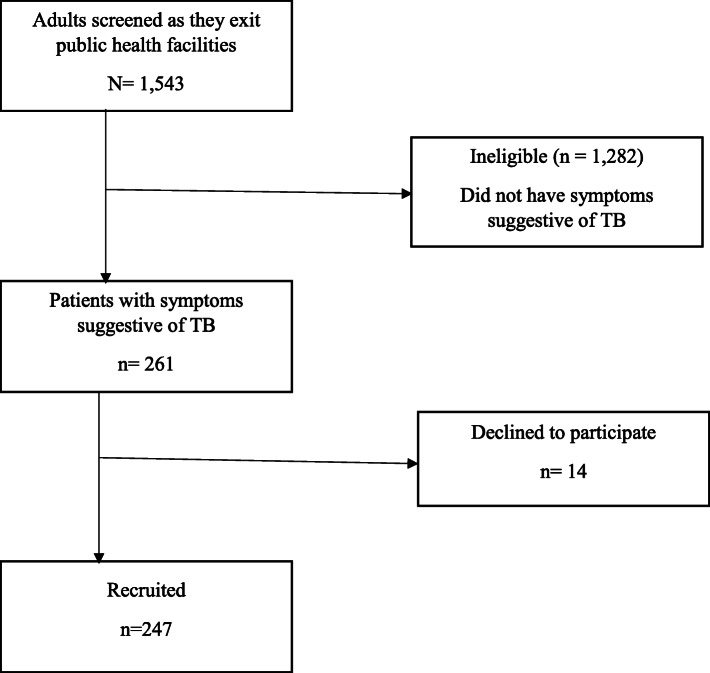
Study flow diagram

From April 2018 to June 2018, we screened 1543 adults upon exiting public health facilities for eligibility of whom 261 met our inclusion criteria (Fig. 1). Of this, 14 (5%) declined enrollment and 247 (95%) were enrolled.

The mean ± SD age of the participants was 35.1 ± 11.5 years, 161(65.2%) were female, 120 (48.6%) had attained primary education and 93 (37.6%) were in full-time employment. Just over half (*n* = 132,53.4%) of the participants were enrolled from the ART clinic, and 128 (51. 8%) resided in urban areas.

Majority (191/247, 77.3%) of the patients had cough and most (116/191, 60.7%) of them had had cough for between 2 and 4 weeks. Only 23/247(9.3%) had the four classical constitutional symptoms of TB. Majority (226/247, 91.5%) of the patients knew their HIV status; with nearly two-third (144/226, 63.7%) being HIV positive and 139/144 (96.5%) were on ART.

Table 1 summarizes the socio-demographic and clinical characteristics of the patients.

**Table 1:** Socio-demographic and clinical characteristics of the respondents

Characteristic	Frequency *N* = 247	Percentage (%)
Entry point		
ART Clinic	132	53.4
OPD	115	46.6
Residence		
Urban	128	51.8
Rural	119	48.2
Sex		
Female	161	65.2
Male	86	34.8
Age		
18–29	93	37.65
30–39	74	29.96
40–49	51	20.65
50 and above	29	11.74
Education level		
None	31	12.6
Primary	120	48.6
Secondary	82	33.2
Tertiary	14	5.7
Employment status		
Employed full-time	93	37.6
Employed part-time	55	22.3
Unemployed	69	27.9
Student	6	2.4
Other^a^	24	9.7
Marital status		
Married	69	27.9
Cohabiting	69	27.9
Single	53	21.5
Divorced	42	17.0
Widow	14	5.7
Distance to health facility		
< 1 km	33	13.4
1-5 km	93	37.6
< 5 km	121	49.0
Tuberculosis Symptoms		
Cough	191	77.3
Duration had the cough (*n* = 191)		
Less than 2 weeks	13	6.8
2 weeks to 4 weeks	116	60.7
> 4 weeks	62	32.5
Persistent fevers	107	43.3
Duration has had the persistent fevers (*n* = 107)		
Less than 2 weeks	22	20.6
2 weeks to 4 weeks	65	60.7
> 4 weeks	20	18.7
Night sweats	95	38.5
Duration of excessive sweats (*n* = 95)		
Less than 2 weeks	11	11.6
2 weeks to 4 weeks	35	36.8
> 4 weeks	49	51.6
Unintentional weight loss	95	38.5
Duration of unintentional weight loss (*n* = 95)		
Less than 2 weeks	17	17.9
2 weeks to 4 weeks	36	37.9
> 4 weeks	42	44.2
Patients who knews their HIV status	226	91.5
HIV Positive	144	63.7
HIV positive on Anti-retroviral therapy	139	96.5
Patients who were referred to Health facility	31	12.5
Reason for visiting the facility		
Getting Anti-retroviral drugs	109	44.1
Get treatment for cough	86	34.8
Others	52	21.1

Other^a^ (Farmer, commercial motorcycle transporters, Business, casual laborer).

### Missed opportunities for TB investigation

Of the 247 enrolled study participants, 138 (55.9%) had at least one symptom suggestive of TB disease but were not offered sputum and/or CXR investigations. Overall, 160/247 (64.8%) reported TB related symptoms to health workers. Less than half (103/247, 41.7%) of the participants were asked by the treating clinician to provide a sputum sample for microscopy and only 19/247 (7.7%) were offered a request for CXR examination. Another 13/247 (5%) were asked to provide both sputum for microscopy and CXR.

### Factors associated with missed opportunities for TB investigation

Only duration of cough (APR; 0.69, 95%CI; 0.56–0.86, *p* < 0.001) and not informing the health worker about TB symptoms (APR; 1.68, 95%CI; 1.36–2.08, *p* < 0.001) were significantly associated with missed opportunities of TB investigation (Table 2).

**Table 2:** Factors Associated with Missed opportunities of TB investigations

Variable	Missed Opportunities		PR (95%CI)	APR (95%CI)	*P*- value
	No (***n*** = 109) Yes (***n*** = 138)				
Sex					
Male	45 (42.3)	41 (29.7)	1.0		
Female	64 (58.7)	97 (70.3)	1.26 (0.98–1.63)	1.26 (0.99–1.61)	0.101
Age					
18–29	38 (34.9)	55 (39.9)	1.0		
30–39	32 (29.4)	42 (30.4)	0.96 (0.74–1.25)	1.20 (0.93–1.56)	0.157
40–49	27 (24.8)	24 (17.4)	0.80 (0.57–1.11)	0.92 (0.67–1.27)	0.615
50 and above	12 (11.0)	17 (12.3)	0.99 (0.70–1.41)	1.27 (0.87–1.87)	0.220
Education level					
None	17 (15.6)	14 (10.1)	1.0		
Primary	52 (47.7)	68 (49.3)	1.25 (0.83–1.91)	1.23 (0.83–1.84)	0.301
Secondary and above	40 (36.7)	56 (40.6)	1.29 (0.85–1.97)	1.20 (0.80–1.77)	0.380
Married					
Yes	58 (53.2)	80 (58.0)	1.0		
No	51 (46.8)	58 (42.0)	0.92 (0.73–1.15)	0.93 (0.75–1.17)	0.545
Cough duration					
Less than 2 weeks	16 (14.7)	53 (38.4)	**1.0**		
2 weeks and more	93 (85.3)	85 (61.6)	**0.62 (0.51–0.76)**	**0.69 (0.56–0.86)**	0.001*
Persistent fever duration					
Less than 2 weeks	62 (56.9)	100 (72.5)	1.0		
2 weeks and more	47 (43.1)	38 (27.5)	0.72 (0.55–0.95)	0.83 (0.611–1.12)	0.224
Number of symptoms					
One	30 (27.5)	64 (46.4)	1.0		
Two	39 (35.8)	49 (35.5)	0.82 (0.65–1.03)	0.99 (0.76–1.29)	0.949
Three	27 (24.8)	15 (10.9)	0.52 (0.34–0.81)	0.70 (0.45–1.10)	0.122
Four	13 (11.9)	10 (7.25)	0.64 (0.39–1.04)	0.89 (0.55–1.45)	0.642
Informed health worker of TB symptoms					
Yes	91 (83.5)	69 (50.0)	1.0	1.0	
No	18 (16.5)	69 (50.0)	**1.83 (1.49–2.26)**	**1.68 (1.36–2.08)**	< 0.001*

*PR Prevalence ratio, *APR* Adjusted prevalence ratio

## Discussion

TB is a preventable and curable infectious disease that continues to infect and claim the lives of a significant number of individuals globally [1]. Early identification of symptoms and appropriate investigations are important for early diagnosis and institution of anti-TB regimen. TB case detection rates has remained low especially in the developing world, including Uganda [1]. The present study was conducted to determine patient factors associated with missed opportunities in TB investigation at public health facilities.

Our study reports a high prevalence of missed opportunities for TB investigations. However, this was relatively lower than what was reported during the Uganda national prevalence survey which stood at 84% [7]. The proportion of patients with symptoms suggestive of TB who were not asked by health workers to provide sputum samples for examination in this study was relatively lower at about 56% compared to previously published studies conducted in Uganda, Ghana, and South Africa reporting a relatively higher proportions ranging from 75 to 90% [15–17] . The observed differences could be attributed to the improved knowledge and vigilance by the health workers through different interventions such as facility-based trainings and mentorships by the government and its partners. However, putting this together, the proportion of missed opportunities for TB investigation is still high suggesting existing gaps in the TB case finding initiatives. Interventions to enhance TB case finding at the health facility need to be implemented.

Of further interest, our study found that reporting symptoms to health worker was associated with having sputum samples sent for TB investigation and/or CXR examination done. Similar findings were reported in South Africa where reporting symptoms to health workers was one of the strongest factors associated with having sputum samples sent for TB investigations [8]. This suggests that patients who had a positive symptom screen for TB were more likely to undergo TB evaluation. Therefore, there is need for health workers to actively screen for TB at health facilities using the standardized TB symptom screen tool.

Furthermore, our study found that patients with cough of duration of 2 weeks or more were about 30% less likely to miss TB investigation compared to those with cough of less than 2 weeks duration. This finding was not in agreement with a study in South Africa where duration of cough did not result into a statistically significant association with TB investigation [9]. This may suggest that patients who meet criteria for presumptive TB but have cough less than 2 weeks are likely to be miss TB evaluation. Moreover, a significant proportion of our study participants were HIV positive who should be screened for TB regardless of the duration of cough. Health workers should be sensitized to adhere to the TB screening and diagnosis protocols that recommend TB evaluation for all patients who meet the WHO predefined criteria.

Our study has some limitations. We used a cross sectional study design that mainly depended on self-reported responses that could be affected by recall bias. Additionally, this study design does not allow for inference for causality. Also, we focused majorly on two entry points at the public health facility –outpatient department and HIV care clinic, which may affect generalizability of our findings at the public health facility. Using the WHO predefined criteria also limited our study from investigating missed opportunities in the diagnosis of extra-pulmonary TB. Furthermore, we didn’t ask whether some patients had been evaluated using urine LAM as one of the TB investigations since it wasn’t widely available at the time. Consequently, this could have limited us from identifying some patients who could have been evaluated using urine LAM technique. Lastly, more females than males were included despite the fact that in Uganda more males have TB than females. This may mean that more males missed opportunities for TB investigation by simply not presenting to health care facilities. However, we report important gaps in facility-based investigations for TB disease in routine clinical practice that merits further research into clinician and patient factors that limits timely TB diagnosis.

## Conclusion

The study showed a high proportion of the patients with symptoms suggestive of TB disease did not have sputum and/or CXR requested for TB investigation, which translates into a high prevalence of missed opportunities for TB investigations in these clinics. Patients who did not inform health workers about their symptoms and those that had shorter duration of cough were more likely to miss TB investigations, which highlights gaps in implementing TB screening and diagnosis protocols by clinicians in public health facilities. While it is important that patients should be empowered to report symptoms, health workers need to proactively implement the WHO TB symptom screen tool with fidelity in order to explore all TB symptoms among patients.

## Data Availability

The datasets used during this study are available from the corresponding author on request.
